# Dorsiflexion Specific Ankle Robotics to Enhance Motor Learning After Stroke: A Preliminary Report

**DOI:** 10.21203/rs.3.rs-4390770/v1

**Published:** 2024-06-25

**Authors:** Anindo Roy, Bradley Hennessie, Charlene Hafer-Macko, Kelly Westlake, Richard Macko

**Affiliations:** University of Maryland, College Park; NextStep Robotics; NextStep Robotics; University of Maryland, Baltimore; NextStep Robotics

**Keywords:** Stroke, Hemiparetic gait, Foot drop, Robotics, Locomotor training, Task-specific training

## Abstract

**Background:**

Robotics has emerged as a promising avenue for gait retraining of persons with chronic hemiparetic gait and footdrop, yet there is a gap regarding the biomechanical adaptations that occur with locomotor learning. We developed an ankle exoskeleton (AMBLE) enabling dorsiflexion assist-as-needed across gait cycle sub-events to train and study the biomechanics of motor learning stroke. This single-armed, non-controlled study investigates effects of nine hours (9 weeks × 2 sessions/week) locomotor task-specific ankle robotics training on gait biomechanics and functional mobility in persons with chronic hemiparetic gait and foot drop. Subjects include N = 16 participants (8 male, 8 female) age 53 ± 12 years with mean 11 ± 8 years since stroke. All baseline and post-training outcomes including optical motion capture for 3-D gait biomechanics are conducted during unassisted (no robot) over-ground walking conditions.

**Findings:**

Robotics training with AMBLE produced significant kinematic improvements in ankle peak dorsiflexion angular velocity (°/s, + 44 [49%], p < 0.05), heel-first foot strikes (%steps, + 14 [15%], p < 0.01) toe-off angle (°, + 83[162%], p < 0.05), and paretic knee flexion (°, + 20 [30%], p < 0.05). Improvements in gait temporal-spatial parameters include increased paretic step length (cm, + 12 [20%], p< 0.05), reduced paretic swing duration (%GC, −3[6%], p < 0.05), and trend toward improved step length symmetry (−16 [11%], p = 0.08). Functional improvements include 10-meter comfortable (m/s, + 13 [16%], p < 0.01) and fastest (m/s, + 13 [15%], p<0.01) walking velocities, 6-minute timed walk distance (m, + 16 [19%], p < 0.01) and Dynamic Gait Index scores (+15 [15%], p < 0.01). Subjects’ perceived improvements surpassed the minimal clinically important difference on the Stroke Impact Scale (SIS) mobility subscale (+11 [19%], p < 0.05).

**Conclusions:**

AMBLE training improves paretic ankle neuromotor control, paretic knee flexion, and gait temporal-distance parameters during unassisted over-ground walking in persons with chronic stroke and foot drop. This locomotor learning indexed by an increase in volitional autonomous (non-robotic) control of paretic ankle across training translated to improvements in functional mobility outcomes. Larger randomized clinical trials are needed to investigate the effectiveness of task-specific ankle robotics, and precise training characteristics to durably improve gait, balance, and home and community-based functional mobility for persons with hemiparetic gait and foot drop.

**Clinical trial identifier:**

NCT04594837.

## INTRODUCTION

Hemiparetic gait (HPG) is the leading neurological deficit that persistently impairs mobility function for persons that have suffered a stroke [1]. The defining elements of HPG are reduced cadence, increased swing time on the paretic side, increased stance time on the non-paretic side, and step length asymmetry [2–3]. Furthermore, HPG presents a broad range of neurological severities, and often includes impaired ankle motor control, sub-optimal foot landing, and reduced dynamic balance; all of which can further impair function and safety [4–5]. Foot drop, present in approximately 30 percent of stroke survivors, is on the more severe end of the spectrum of chronic HPG deficits and is associated with reduced gait efficiency and high fall risk [6]. However, foot drop after stroke never exists as an isolated entity. Other elements of HPG and a variety of compensatory gait strategies superimpose upon foot drop to influence the overall biomechanical characteristics of gait and the functional mobility and fall risk outcomes. Hence, foot drop has proven a difficult therapeutic target, primarily relegated to assistive devices in practice.

Robotics has emerged as a promising avenue for gait re-training after stroke, even for individuals with otherwise refractory paretic foot-drop remaining long after all conventional physical therapy has ended [7]. Our prior randomized studies show that task-specific training using a wearable impedance control exoskeleton (“Anklebot”, [8]) for treadmill-based gait training, as opposed to the same dose of deficit-focused seated computer-video interfaced paretic ankle joint training, is significantly more effective to reverse foot drop and durably improve volitional unassisted over-ground gait for persons with chronic hemiparetic stroke [9]. As predicted, task-specific treadmill training after stroke was substantially more effective in improving unassisted over-ground gait than deficit-focused, paretic ankle seated video game training, which is better for improving isolated ankle motor control, but not walking.

Despite our findings and those of others that task-specific neuro-robotics can improve selected elements of hemiparetic gait, there is a gap regarding the broader nature of functional and biomechanical adaptations that occur with training. Most of the research has focused on gait velocity, foot landing, and gait temporal-distance parameters. Moreover, our prior studies were limited by the form factor of the laboratory-grade predecessor device, with relatively heavy weight and tethering cables restricting training to seated or treadmill based modalities. Therefore, we developed a wearable powered and blue-tooth operatable ankle exoskeleton (AMBLE) with deficit severity adjustable impedance control for human robotics cooperative learning. AMBLE use during treadmill and/or overground walking training enables further exploration of the nature of hemiparetic gait adaptations that may be mediated by task-specific training across specific portions of the gait cycle. Development of AMBLE also mitigated barriers to clinical adoption, including integration into clinical work flow, existing reimbursement pathways, and access through affordability.

In this study we used AMBLE to isolate and precisely control the task-specific nature of the robotics mediated locomotor learning stimulus, herein with a focus on re-training ankle dorsiflexion across sub-phases of the swing phase of gait and initial contact. Specifically, dorsiflexion during swing starts with a dorsi-flexion rise phase following toe-off, a hold phase during mid-swing, and the ankle dorsiflexion aided by tibialis anterior contraction upon initial contact to soften the landing, thereby avoiding the pathologically elevated ground reaction forces of foot slap. AMBLE is designed to assist as-needed across these three dorsi-flexion sub-phases, with initial support calibrated to participants’ baseline gait, and progressed toward volitional (vs. robot) control according to their ongoing abilities, as indicated by the step-by-step and cumulative measure of kinematic autonomy. This preliminary report investigates the effects of robot-aided ankle training during walking on functional mobility outcomes, and gait biomechanics in persons with chronic HPG and foot drop. Subjects with chronic HPG were selected to isolate the robotic assisted task specific locomotor training mediated from early natural neurological recovery from stroke. While the anticipated finding was improved foot-drop specific outcomes, we posit that robotics mediated training would extend to proximal lower extremity joints, to improve unassisted overground gait and functional mobility outcomes.

## METHODS

### Study Inclusion/Exclusion Criteria.

Inclusion criteria consisted of: (i) unilateral ischemic or hemorrhagic stroke, (ii) > 6 months post-stroke, (iii) age > 18 years, (iv) medical clearance, (iii) residual hemiparesis of the lower extremity that included symptoms of foot-drop (see criteria below*), (iv) completed all conventional therapy, (v) ability to ambulate at least 5ft without an AFO and with no more than minimal contact assistance, (vi) ability to follow a 3-step command. *Dorsiflexion deficits: For the purposes of study inclusion, dorsiflexion (DF) deficits were defined based on active range of motion (AROM) and strength. Mean maximal DF AROM measured in duplicate by trained staff using goniometry with subjects seated on plinth with knees at edge, lower legs dangling at 90°. DF deficit criterion was met when subjects were unable to reach zero degrees (foot parallel to floor, perpendicular to shank). Manual muscle testing was performed in the same seated position with inclusion criteria between grade 1/5 (trace) to 4/5 (reduced strength) in DF. Though AFO use is consistent with DF deficits, there are many factors (e.g. compensatory gait strategies) that influence this status. Exclusion criteria consisted of: (i) cardiac history of unstable angina, recent (< 3months) myocardial infarction, congestive heart failure (NYHA category II), or hemodynamically significant valvular dysfunction, (ii) hypertension that is a contraindication for routine physical therapy (>160/100 on two assessments), (iii) medical history includes recent hospitalization (< 3 months) for severe medical disease, symptomatic peripheral arterial occlusive disease, orthopedic or chronic pain conditions that significantly alter gait function, pulmonary or renal failure, or active cancer, (iv) history of non-stroke neuromuscular disorder restricting gait, (v) aphasia or cognitive functioning that confounds participation, defined as unable to follow 2-step commands or judgment of the clinician.

### Study Design and Procedures.

This was a single-arm pilot study in 16 subjects with chronic stroke and foot drop. Subjects underwent screening to ensure study criteria was met, which included review of medical and neuro-imaging records, medical and neurologic exams. Baseline neurologic exam characterized deficit severity and stroke subtype based on clinical and radiographic data. Exams screened for medical and cardiopulmonary safety for walking with the AMBLE device. Eligible subjects received 9 weeks of AMBLE therapy (2 sessions per week, ~ 40 minutes duration per visit).

### Assessments and Outcomes.

To establish efficacy in this chronic stroke population, we had participants serve as their own pseudo-controls by double baseline testing with two tests conducted one week apart. All testing was conducted at NextStep Robotics. Testing included the NIH Stroke Impact Scale (SIS version 3.0); timed 10-meter walk and 6-minute walk tests (6MWT); Modified Dynamic Gait Index (DGI); and gait biomechanics acquired using motion capture system (OptiTrak^™^) during over-ground walking. The use and type of assistive device and/or ankle orthoses were recorded at each visit. Post-testing was conducted after the 18 study sessions were completed and included the same assessments as those administered during baseline tests. For variables of gait biomechanics, a mean of all pooled steps from at least 3 trials. Two derived variables i.e., step length *(SI*_SL_**)** and stance duration symmetry *(SI*_*SD*_**)** indices were calculated as:

1 $$\text{\%}SI_{SL}=100\times \left(1-\frac{SL_{P}}{SL_{NP}}\right),\text{\%}SI_{SD}=100\times \left(\frac{SST_{P}}{SST_{NP}}\right)$$

where *SL* and *SST* denote step length and single support stance duration, respectively, and subscripts “*P*” and *“NP”* denoting the paretic and non-paretic sides, respectively.

### Human Autonomy Index.

A robot-measured outcome, the Human Autonomy Index (HAI), was computed for each training visit. The HAI is defined as the total time spent by the paretic ankle within pre-defined “normative” range within which no assistance is rendered (vs. outside the ranges in which springy assistance is provided) i.e.,

(2) $$\text{\%}HAI=\frac{\sum N\left(t_{i}\right)}{N_{SW}},N\left(t_{i}\right)=1\;\text{if}\;B\left(t_{i}\right)\leq \theta \left(t_{i}\right)\leq T\left(t_{i}\right),t_{TO}\leq t_{i}\leq t_{SW}$$

where *N* is the number of samples for which the ankle angle *θ (t*_*i*_**)** is within the normative range, *N_SW_* is the total number of samples until time to peak, *B (t*_*i*_**)** and *T (t*_*i*_**)** are the bottom and top profiles around the reference trajectory *θ*_*ref*_, and *t*_*TO*_ and *t*_*sw*_ are the toe-off and time-to-peak instants, respectively.

### Apparatus.

**Figure 1:**
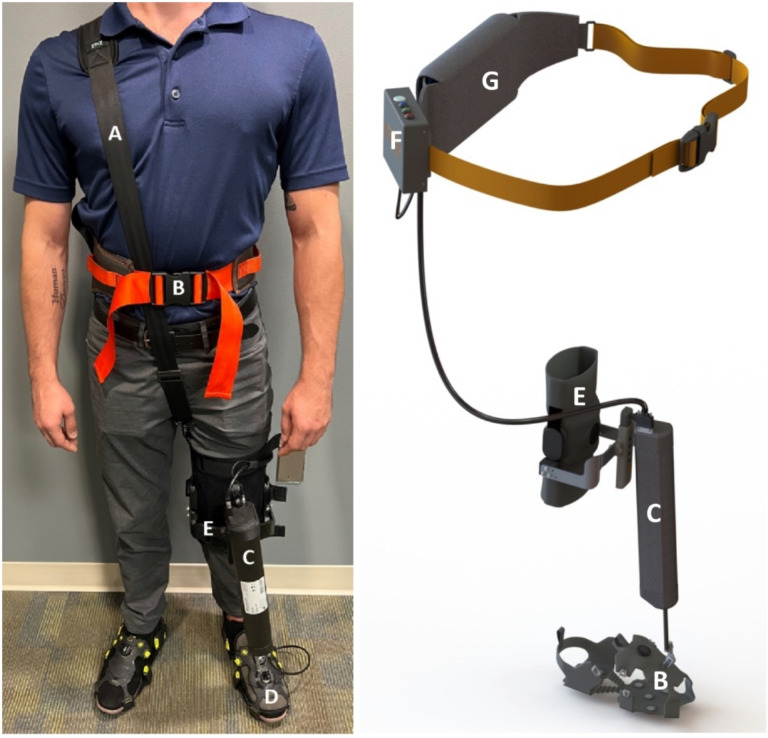
The AMBLE device and its key sub-systems. **A**: shoulder strap, **B**: gait belt, **C**: Actuator unit, **D**: Sensor crampon, **E**: knee brace, **F**: control box, **G**: battery pouch.

AMBLE is a Class I body-worn, powered exerciser intended to restore ankle function during walking (Fig. 1). It is worn unilaterally on the affected side, provides active mechanical assistance to facilitate swing dorsiflexion, and is customized to each user’s volitional ankle strength and range of motion, to allow users to engage in treadmill or indoor over-ground walking exercises.

#### Design and Hardware.

Specifically, AMBLE is a one degree-of-freedom (1-DOF) wearable robot, back-drivable with low intrinsic mechanical impedance, that weighs less than 1.4 kg. It allows normal ROM in all 3 DOF’s of the foot relative to the shank during walking overground or on a treadmill. The AMBLE provides actuation in one of the ankle’s three DOF’s, namely dorsiflexion via a single linear actuator. AMBLE allows 25° of dorsiflexion and 35° of plantarflexion free ROM at the ankle, limits that are near the maximum range of comfortable motion for normal subjects and beyond what is required for typical gait. The AMBLE can deliver a continuous net torque of approximately 20Nm in dorsiflexion, which is higher than required to position the foot in dorsiflexion during mid-swing. It has low friction (~ 6.6N) and inertia (total of 1.4kg at the foot) to maximize the back-drivability. The AMBLE is battery powered (2600mAh, Inspired Energy) and actuated by a single brushless DC motor (Maxon EC-i 40, Maxon, Switzerland), capable of generating 0.224Nm nominal and 2.1Nm stall torque, respectively, which is amplified (226m^−1^) and transmitted to the foot piece via a traction drive consisting of a linear screw actuator (NextStep Robotics, MD). AMBLE includes a sensorized crampon that detects gait events, information that is relayed to the controller for precisely timed as-needed actuation to aid swing dorsiflexion.

#### Sensing and Control.

**Figure 2:**
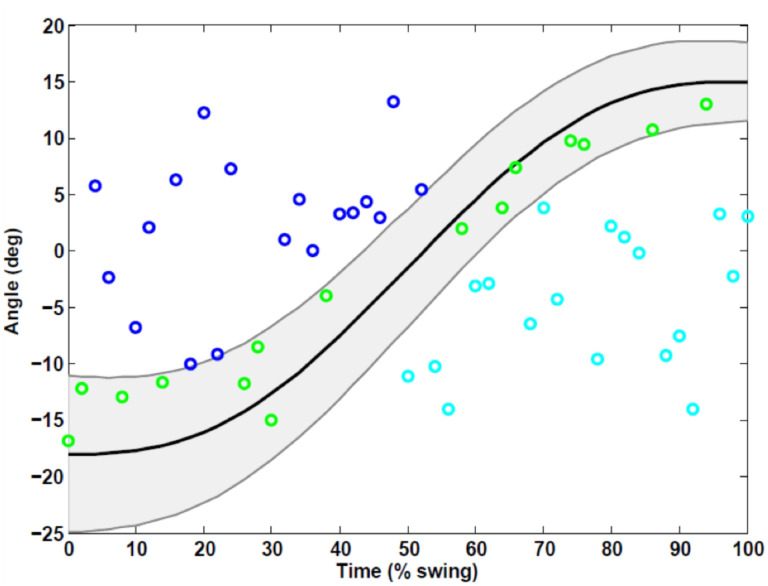
Concept of assistance as-needed illustrated by exemplar data from a single subject from a single step during the swing phase. Top and bottom trajectories (—) are created around the reference trajectory (solid —) to render a no-assist region (grey area). Only when the ankle position is either below the bottom (**c**) or above the top (**o**) edges does the subject receive active assistance.

AMBLE employs a linear absolute magnetic encoder (LA11, RLS, Komenda, Slovenia) with a resolution of 3.9μm mounted on the traction drive. The linear excursion measured by the encoder is used as feedback to the controller, to estimate ankle angle in dorsi-plantarflexion using a simple mathematical model of the shank-ankle-foot system. Torque is measured by an empirical, linear model relating motor torque to pulse width modulation (PWM) duty cycles. For control, the AMBLE employs a simple impedance controller with a programmable reference position, a programmable proportional gain (approximating a controllable torsional stiffness), and a programmable derivative gain (approximating a controllable torsional damping in parallel with the stiffness). AMBLE utilizes a minimum jerk trajectory as its reference and provides active assistance as-needed by creating a force field around the reference. In this schema, dynamic trajectories are created around the reference to reflect “normative” ankle movements and no forces are generated by the device within these dynamic boundaries to promote autonomy (Fig. 2). For gait training, AMBLE employs a custom control-and-command software app on an iPad, which is used to set and progress therapy inputs customized to each patient’s deficit severity and needs.

#### Safety and Usability.

AMBLE failure analysis considered don and doff times, as well as hardware failures. During don and doff, the patient remains seated eliminating any potential for falls. In case of emergency, the device can be removed from the patient in less than 30sec. The software continuously monitors torques, velocities, and displacements and disables the system in case preestablished limits are exceeded or in case of critical component malfunctions (e.g., linear encoder). Furthermore, the traction drive operates as a mechanical “fuse,” and it slides above preset torque values (96–116N). Additionally, a “stop” button on the control box is available to manually stop the device. Excluding the time needed to determine the patient’s knee brace and shoe sizes, the donning process requires < 5min by a single clinician. Multiple sizes of the knee brace and crampons are available to accommodate individuals with different anthropometric dimensions and for optimal comfort.

### Intervention.

Training sessions were conducted at NextStep Robotics by two exercise physiologists who were trained in the use of the AMBLE device. Participants received a total of 18 supervised treadmill (~ 20min) and over-ground (~ 20min) walking sessions while receiving assistance as-needed from the AMBLE device across a 9-week time period. Individuals that missed three consecutive training sessions were excluded. Each visit required ~ 1 hour that included: (1) pre-gait activities, which occur without the robot (don, calibration), 2) treadmill and over-ground gait with the AMBLE device in active assist mode; 3) Post-gait activities, which occur without the robot (doff). Pre-training activities consisted of checking user dimensions for device sizing (knee brace, crampon, shoulder strap). Any subject whose anthropometric characteristics prevent fit (e.g., foot size outside the range of available crampon sizes) were excluded at this stage. Trainers remove any AFOs worn by patients prior to donning and record the time it takes to put the device on the user. Additionally, on the first day of training, after fitting the robot to the participant, subjects walk for 10 steps with the robot in “evaluation” mode to acquire measures of unaided gait function that are used as therapy inputs by the trainer to customize the reference trajectory (volitional peak swing DF angle, rise speed, and hold time). These initial 10 seps act as a baseline calibration for the robot to provide an initial ankle angle trajectory. The between-session use and type of assistive device and/or ankle or other orthoses were recorded at each visit.

Both treadmill and over-ground walking consisted of a scripted recommendation for subjects to walk at their comfortable self-selected pace. During over-ground walking, subjects walked at their comfortable self-selected pace, completing a total of ~ 20min. Subjects were allowed to use their assistive devices (AD) for safety, if needed. Treadmill training consisted of walking on an instrumented treadmill (Biodex GT3) at self-selected comfortable speed with handrail support, completing a total ~ 20min. Subjects were informed of the duration of their walking time with a goal of a total of 40 minutes active supervised walking, as tolerated, but not to exceed 45 minutes. A Polar Heart Rate Monitor was used during the supervised walking with the maximal allowed heart rate during walking < 50% of heart rate reserve, based upon the formula of Karvonen, adjusted for any chronotropic medication usage (e.g. beta blockers), as by convention. The purpose was to assure that participants are maintained at a low aerobic exercise intensity, as defined according to the criterion of the American College of Sports Medicine (ACSM), and consistent with the levels of aerobic intensity that are typically encountered during routine physical therapy after stroke. The time spent in walking and resting were recorded as total therapy and total session times, respectively. Post-training activities consisted of doffing the device and recording the doff time as well as visual inspection of the paretic lower extremity for any adverse events (AEs), such as fatigue, cardiopulmonary issues (e.g., shortness of breath, dizziness), pinch points, musculoskeletal discomfort (e.g., joint pain), skin irritation, chafing, discomfort due to device slippage or weight, etc. Further, device malfunctions and clinician use errors were recorded throughout the study.

### Statistical Analysis.

Longitudinal analysis included computing the differences in outcomes between baseline and post testing sessions. Data from the first and last steps during walking assessments were not included in the analyses to eliminate any confounding effects due to partial foot contacts at the extremes of the motion capture area. For patient reported outcomes (SIS ADL and Mobility scores), only the 10-item ADL/IADL and the 9-item mobility scores were compared from pre- to post-test. HAI data, as obtained from each training session, were averaged across all subjects, then averaged across three bins of 6 visits. Comparisons were made between the first and last 6-visit bins. All continuous data were checked for normal distribution using the Kolmogorov-Smirnov Test. If normally distributed, paired t tests were used to compare the means at baseline and post testing, otherwise the Wilcoxon Rank Sum Test was used. SIS results pre and post-training were compared using the Wilcoxon Rank Sum Test. The significance level was set at alpha 0.05.

## RESULTS

**Figure 3:**
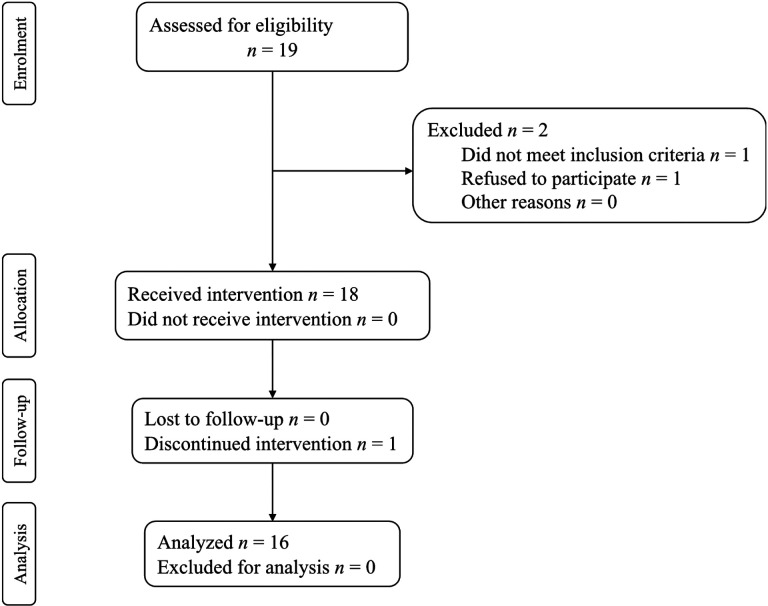
Consort flow chart.

This interim safety and effectiveness analysis of use of AMBLE in chronic stroke participants with foot drop is part of a larger, ongoing NINDS Phase II clinical trial and has 30 participants enrolled so far, of which 16 participants have completed all planned visits and activities and are included in the analysis of the clinical endpoints. The consort flow chart is shown in Fig. 3. All outcomes at each endpoint were normally distributed, so paired t-tests were used for comparison across endpoints.

### Baseline Characteristics.

Baseline demographics of completers are summarized in Table 1. Subjects on average were 53 years of age, >10 years post-stroke with foot drop, and limited community ambulators (0.4–0.8m/s). Individually, a majority were limited community ambulators and a majority customarily used passive AFOs (62.5%) or walking aids such as single-point or quad canes (37.5%). A majority (> 60%) had mild impairments in ankle strength (MMT grade 4/5). For each training session, the HAI data were averaged across all subjects, then averaged across three bins of 6 visits. Comparisons were made between the first and last 6-visit bins (Table 2).

### Device Usage/Safety/Reliability.

Subjects were exposed to ~ 9.4 hours of walking with the device with an average cumulative session duration of ~12 hours. On average, the group walked >1100 steps per visit, which is ~ 40% of an average person’s daily step count [28–29]. There were zero device-related adverse (AEs) or serious adverse events (SAEs). There were also no injuries to the operators. Device malfunctions were reported to have occurred in <1% of study visits and were related to fuse-initiated battery trips. Malfunctions were resolved by restarting the device. None of these device malfunctions resulted in AEs or SAEs nor did they result in the need to alter the originally planned gait training activities. Mild, study-related risk/discomfort resulted from to sub-optimal fitting i.e., one subject the smallest crampon size was not sufficiently snug) while another subject had pressure and/or pinch point on shin that resolved its own and did not require medical intervention.

### Gait Biomechanics and Joint Kinematics.

Improvements were observed in key paretic ankle motor control outcomes namely (Table 3), peak dorsiflexion angular velocity (°/s, + 44[49%], p < 0.05) and heel-first foot strikes (%steps, + 14[15%]. This was accompanied by significant increase paretic knee flexion (°, + 20[30%], p < 0.05). Although not a foot drop outcome, a significant increase was also observed in the toe-off angle (°, + 83[162%], p < 0.05). Associated with these changes in joint kinematics were significant increases in paretic step length (cm, + 12[20%], p < 0.05) and reduction in paretic swing duration (%GC, −3[6%], p < 0.05) along with higher step length symmetry (−16[11%], p = 0.08) trending towards significance.

### Functional Outcomes.

**Figure 4:**
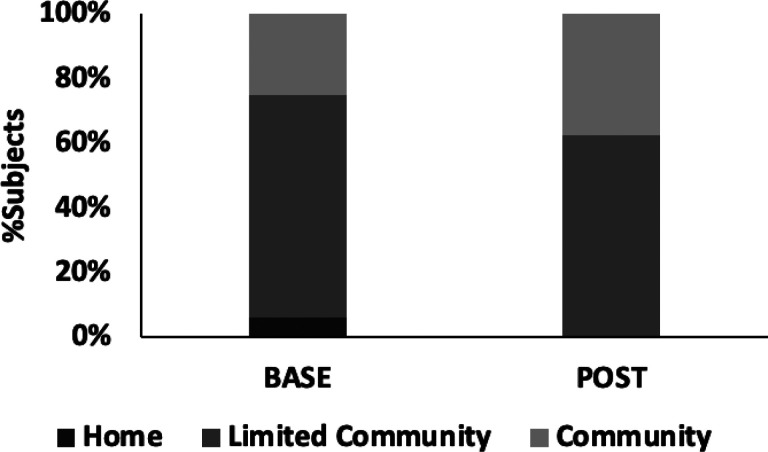
Distribution of subjects across ambulation categories [35] (Home: <0.4m/s, Limited Community: 0.4–0.8m/s, Community: >0.8m/s) between baseline and post-testing.

Subjects showed statistically significant but modest improvements in their 10-meter overground gait speed, under both comfortable (m/s, +13[16]%, p < 0.01) and fastest (m/s, + 13[15%], p < 0.01) walking conditions. As a group, participants trended towards a higher ambulation category i.e., from limited community (LC) ambulator (0.71m/s) toward community ambulator (0.78m/s). Individual changes in ambulation category were also observed in 3 participants: one from home (<0.4m/s) to limited community ambulator (0.4–0.8m/s) and two from limited to community ambulator (> 0.8ms) (Fig. 4). Eleven subjects (69%) showed a small meaningful change in gait speed (> 0.05m/s but < 0.1m/s) while six subjects (37%) showed large meaningful changes (> 0.1m/s) and of those, 50% also improved their ambulation category.

Significant improvements were also observed in the DGI score (+ 15[15%], p < 0.01) and the 6-minute timed walk distance (m, + 16[19%], p < 0.01). Notably, the average change in DGI and 6MWT distance were greater than the corresponding minimum clinically important difference (MCIDs) or the minimum detectable change (MDC) (as appropriate), while the change in peak knee flexion trended towards its corresponding MCID (Table 4). At the individual level, 62% and 37% of subjects showed clinically meaningful increases in their DGI scores and 6MWT distance. However, change in walking speed, both at comfortable and fast conditions, were modest, being below the corresponding MDC for these clinical outcomes.

### Patient-Reported Outcomes.

Associated with functional improvements were significant increases in the patient-reported score on the SIS mobility subscale (+11[19%], p < 0.05), which was also greater than the corresponding MCID. The average change score on the SIS ADL/IADL subscale (+15[32%]) was below the MCID but trended towards statistical significance (p = 0.06). Within subjects, 31% reported higher than clinically meaningful changes in both ADL as well as mobility scores.

### Human Autonomy Index.

**Figure 5:**
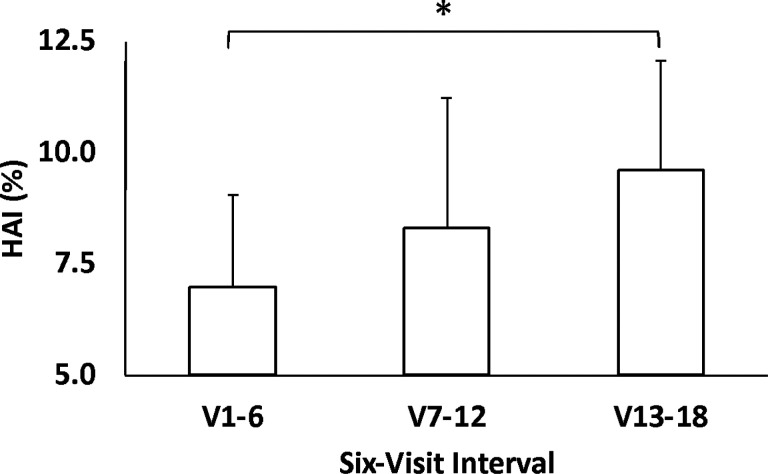
Progression of average HAI as a function of training visits, the latter grouped into six-visit bins.

As a group, subjects trended toward significant improvements in HAI between the first and the last six-interval bins (+ 44[47%], p = 0.05), reflective of a higher incidence of volitional ankle movements during assisted trials (Fig. 5).

## DISCUSSION

AMBLE training exclusively in the dorsiflexion assist-as-needed mode showed preliminary effectiveness in improving multiple biomechanical elements of unassisted over-ground gait and selected functional mobility outcomes in persons with chronic hemiparetic gait and foot drop following stroke. The biomechanical changes include, but are not restricted to, hemiparetic ankle neuromotor control, with improvements up the kinematic chain, and in gait spatio-temporal parameters fundamental to the definition of hemiparetic gait. Improvements in ankle neuromotor control include increased peak dorsi-flexion angular velocity, heel first foot strikes, and toe-off angle; all of which could lessen foot-drop by virtue of improved dorsi-flexion across the gait cycle. Improvements up the kinematic chain include greater paretic knee flexion, but not hip kinematics in this limited sample size study. AMBLE training improved selected gait-temporal distance parameters including paretic step length, paretic swing duration, and this produced a trend toward higher step length symmetry. Notably, these improvements are not short-term carry-over effects from AMBLE training, as all measurements are performed during unassisted over-ground gait (no robot assist) at least 48 hours following the last robotics training session. Further, that locomotor learning is responsible for these improvements is supported by significant and monotonic improvements in human autonomy index across training Collectively, these biomechanical improvements in hemiparetic gait translated into improved functional mobility indexed by improved gait speed, endurance, and stability (i.e., 10m and 6 min walk tests and Dynamic Gait Index scores).

### Relevance of Improved Joint Kinematics

Tripping over an obstacle is one of the biggest causes of falling in the elderly [10–11]. AMBLE participants improved their foot control as demonstrated by greater toe clearance, sufficient to clear typical obstacles encountered at home ranging from <1cm to 8cm [12]. Improvements in ankle control were also evidenced by a higher incidence of heel-first foot strikes. Digitigrade walking, as observed and manifesting as toe-drag in persons with foot drop, increases the external mechanical work performed by the limbs. In particular, this requires a higher energy cost of transport [13] and imposes excessive kinetic burden that increases the risk of knee injury, including but not limited to anterior cruciate ligament (ACL) injuries [14]. Alternately, lateral foot contact is equally unsafe as it can cause ankle sprains via the inversion mechanism, especially given that chronic stroke patients have weak inverter musculature, resulting in greater mechanical compliance in inversion. In contrast, walking with a heel-first strike pattern can reduce the loading forces of the knee joint [14] and has been shown to improve the economy of walking [13]. Participants also experienced significant increases in their peak DF velocity, which has been shown to be a valid and reliable task-specific measure of ankle dorsiflexion during walking [15]. Further, increased knee flexion as evidenced here, is known to facilitate toe clearance to safely clear the ground, further supporting our hypothesis that training effects go beyond the targeted joint. Positive changes in joint kinematics translated to improvements in select measures of gait biomechanics. Specifically, participants evolved a more normative temporal pattern of walking, by increasing their paretic stance while reducing their swing duration. Instability on the paretic limb can cause compensatory shortening of paretic stance time, as this is considered to reflect balance ability [16]. Similarly, leg stiffness due to the impaired paretic limb causes compensatory prolonged swing time [17]. This typical temporal pattern of gait abnormality in hemiparetic patients with prolonged paretic swing time and shortened nonparetic swing has been shown to reflect paretic limb function impairment [18].

### Improvements in Walking Function

Slower speed has been associated with increased risk of falls, even after adjustments for potential confounders and traditional clinical tests of cognition, gait, and balance [19]. In fact, each 10 cm/s decrease in gait speed has been associated with a 7% increased risk for falls and community-dwelling older adults with slow gait speed (< 0.7m/s) demonstrate a 1.5-fold increased risk for falls compared with those with normal speed [19]. While the average increase in gait speed was modest, the group trended towards a higher ambulation category-from limited community ambulator (0.4–0.8m/s)-to community ambulator (> 0.8m/s). Within the group, the largest transition was from limited to full community ambulators (12%). Further, a majority (68%) presented small meaningful changes (> 0.05m/s) while a significant proportion (38%) demonstrated large meaningful changes (>0.1m/s) in walking speed. Participants also improved their dynamic balance trending toward safe ambulators (DGI score) as well as endurance (6MWT distance), with changes in both being higher than their corresponding minimum clinically important differences (MCID’s). At the individual level, a significant proportion improved their dynamic balance (63%) and endurance (38%) by an amount greater than the corresponding MCID’s. These improvements reflect faster and, more importantly, safer walking over longer distances, which is also corroborated by more than 30% subjects self-reporting clinically significant improvements in mobility function (SIS Mobility score).

### Clinical and Mechanistic Implications for Neuromuscular Re-Education

In terms of North American clinical practice, AMBLE may be considered as a therapeutic device targeting neuromuscular re-education. Neuromuscular re-education defined even seven decades ago recognized the misnomer; only the nervous system learns, and thus adaptations are primarily neural [20]. Our results provide evidence that AMBLE produces general improvements in functional mobility (e.g. timed walks, DGI) in the context of specific neurological adaptations to the biomechanics of hemiparetic gait. That progressive increases across training occurred in the Human Autonomy Index, reflecting the human taking over for the robot, implies activity dependent neuroplasticity. The temporal profile of locomotor learning as we report across 9 weeks training cannot be directly compared to assistive devices such as AFO’s or FES, in which outcomes are often measured after prolonged usage, such as a year, or while wearing the device [21–22]. Implications for neural learning vs generalized neuromuscular re-education are quite different for assistive versus therapeutic devices, across different time-frames, and with respect to functional mobility outcomes [23–24]. Our earlier task-oriented training in persons with HPG showed neuroplasticity in convergent subcortical brain networks subserving locomotor control [25]. Our seated ankle robotics training studies revealed cortical sensory-motor re-organization associated with quality and temporal profile of ankle neuromotor learning [26]. However, this study did not investigate mechanisms of neuroplasticity. Further studies are needed to investigate whether exoskeleton mediated locomotor learning can durably improve mobility outcomes and safety for persons with hemiparetic gait across the phases of recovery, and by what adaptive plasticity mechanisms. In terms of clinical interpretation, attention is warranted to the definition of neuromuscular re-education, as we cannot determine precisely which adaptations are central neural, peripheral, or generalized and not specific to locomotor learning in this study.

### Implications for Clinical Translation

Clinical studies using ankle rehabilitation robots have shown improvements in the dorsiflexion angle in the swing phase and propulsion on the paretic side during push-off after a period of robot-assisted ankle rehabilitation training. However, most are case or case control studies with very limited sample sizes [27], limiting evidence-based consensus for their use. Moreover, despite advances in gait robotic technologies in their hardware and manner of control, translation to clinical practice has continued to lag development. Recent commentary from practitioners and rehabilitation researchers have emphasized the need for the next generation of robotic devices to be “agile therapy tools” that enhance, rather than replace, the therapist’s role while encouraging participation and engagement from the patient to maximize motor learning [28]. Such tools should be flexible in empowering PT’s to adapt and intensify patient training (e.g., high intensity training) while also enabling diverse locomotor training modalities without increasing their own labor burden/workload. Further, gait robotics should be capable of accommodating a vast spectrum of pathological gaits and impairment levels. Unlike other devices, AMBLE not only provides highly repeatable, assist as-needed control of rhythmic movements but also addresses the distinct functional needs within the swing phase. Its controller can be programmed as a continuous passive machine to deliver range of motion stretching, AAN for walking exercise, or an assessment tool. Its adaptive timing algorithm [30] ensures step-to-step human-machine synchronicity throughout the gait cycle, thereby allowing safe over-ground and treadmill walking, rendering a versatile locomotor learning tool. AMBLE offers a streamlined don/doff time of under 5 minutes, making it easy to incorporate into existing clinical workflows. It also provides PT’s with highly relevant outcomes such as HAI to characterize locomotor learning and inform customization of therapy. These, and other features are a step toward resolving longstanding barriers that have precluded existing gait robotics from realizing their full potential for clinical translation.

### Study Limitations

This study is limited by small sample size, non-controlled design, restriction to chronic stroke, and lack of durability testing. The small sample size, restriction to chronic stroke, non-controlled design, and lack of durability testing are by design, as this is a preliminary report on a larger clinical trial that includes durability outcomes testing and sub-acute stroke studies. The non-controlled study design in this Phase 2 study for persons with chronic stroke was approved by our National Institute of Health (NINDS) cooperative study advisors following Phase I findings that two weeks training (3x week) using only the weight of the robot (AMBLE worn in the off-state; no actuation) produces no significant biomechanical adaptations. Stability across repeated baseline testing is interpreted in the current report as evidence that the current locomotor learning is mediated by task-specific training with the actuated AMBLE in an otherwise stable neurological cohort. Randomized clinical trials with a reference-treatment control group in persons with sub-acute stroke are ongoing to investigate whether AMBLE improves hemiparetic gait during sub-acute stroke recovery. Regardless, small sample size prevents subset analysis of how persons with different compensatory gait strategies might respond differentially to AMBLE training [3]. Though participant testimonials describe improved functional mobility at home, we did not measure indices of free-living quality and quantity of mobility gains, and thus cannot determine whether laboratory measured gains translate into improved free living mobility and safety.

## CONCLUSIONS

In conclusion, task-specific AMBLE dorsiflexion training improves paretic ankle neuromotor control, paretic knee flexion, and gait temporal-distance parameters during unassisted over-ground walking in persons with chronic hemiparetic stroke and foot drop. These robotics mediated improvements in the biomechanics of hemiparetic gait translate into clinically important improvements in functional mobility, including 6min walk test, DGI, and SIS Mobility Subscale. That the human autonomy index, i.e. proportion of ankle movements generated by the human as opposed to the robot, increased progressively across training provides evidence that these biomechanical and functional benefits are attributable to locomotor learning. Our results must be interpreted with caution as this is a preliminary report with limited sample size and durability outcomes yet to be reported. Larger randomized clinical trials are needed to investigate the effectiveness of task-specific ankle robotics, and the precise training characteristics utilizing this tool that are needed to durably improve gait, balance, and free-living functional mobility for persons that have suffered a disabling hemiparetic stroke.

## Figures and Tables

**Table 1:** Baseline characteristics (N = 16).

ID	Age (yr.)	TPS (mos.)	Gender	Paretic Side	AFO	AD*	MMT	AROM (deg)	Speed (m/s)	Ambulation Category
1	76	266	F	R	Y	Y	1+	−19	0.370	H
2	37	235	F	R	Y	N	4−	−17	0.970	C
4	70	19	M	L	N	4PC	4−	−5	0.460	LC
5	49	36	M	R	N	N	4−	−13	0.929	C
6	68	134	F	R	Y	N	4−	−8	0.550	LC
7	44	14	F	L	N	1PC	4−	−3	0.620	LC
8	34	19	F	L	Y	N	4−	−14	0.699	LC
9	58	86	M	L	N	1PC	4−	−5	0.785	LC
10	30	360	F	L	Y	N	1	0	1.000	C
11	60	233	M	L	Y	1PC	4−	−19	0.844	C
12	55	138	M	R	Y	N	4−	5	0.796	LC
14	47	56	F	L	Y	1PC	1	0	0.483	LC
16	52	95	M	L	N	1PC	2+	−15	0.603	LC
18	39	66	M	R	Y	1PC	0	−25	0.738	LC
20	65	25	F	L	Y	4PC	2−	−5	0.510	LC
21	64	308	M	L	N	1PC	4+	0	0.945	LC

TPS: time post-stroke, AFO: ankle foot orthotic, AD: assistive device, MMT: manual muscle test in dorsiflexion, DGI: dynamic gait index, H/C/LC: home/limited/community ambulator, AROM: active ROM in dorsiflexion, 4PC: quad cane, 1PC: single point cane.

**Table 2:** Comparison of outcomes between repeated baseline tests.

Outcome	Baseline-1	Baseline-2	|Δ(SD)|*	P
DGI (0–24)	16.3 (3.7)	16.3	0.0 (0.8)	1.00
6MWT (m)	282.2 (94.2)	290.7	8.4 (26.5)	0.22
10m Comfortable Speed (m/s)	0.71 (0.20)	0.7	0.03 (0.06)	0.49
10m Fastest Speed (m/s)	0.87 (0.27)	0.9	0.03 (0.1)	0.24
Peak Swing Angle (deg)	0.43 (6.9)	−0.2	0.7 (2.4)	0.27
Maximum Toe Clearance (mm)	63.0 (29.2)	61.2	1.8 (15.3)	0.64
Minimum Toe Clearance (mm)	21.9 (17.0)	22.9	0.9 (5.61)	0.55
Maximum DF Angular Velocity (deg/s)	35.3 (31.6)	38.7	3.4 (15.2)	0.39
Hip Flexion (deg)	35.4 (8.8)	34.3	1.2 (4.0)	0.26
Hip Hike (cm)	8.2 (3.3)	−1.6	0.1 (3.0)	0.89
Hip Abduction (deg)	−7.9 (4.8)	−8.3	0.3 (4.1)	0.78
Knee Flexion (deg)	33.4 (17.6)	34.5	1.1 (5.9)	0.46
Paretic Step length (cm)	40.3 (14.3)	41.2	0.9 (3.3)	0.27
Paretic Swing (%)	40.6 (2.9)	59.5	0.09 (1.8)	0.85
Double Support Stance (%)	23.5 (4.0)	23.2	0.3 (2.1)	0.60
Heel-First Strikes (%steps)	31.3 (43.2)	31.3	0.05 (2.3)	0.93

* Denotes absolute value of difference. 6WMT: six-minute walk test.

**Table 3:** Outcomes at baseline and post-test endpoints (N = 16). Values reported are mean (SE).

Outcome	Baseline Mean (SD)	Post Mean (SD)	Δ(SD)	P
SIS-ADL (0–100)	72.8 (18.2)	79.7 (14.5)	6.9 (13.6)	0.062
SIS-Mobility (0–100)	81.4 (15.1)	88.2 (8.6)	6.8 (10.1)	**0.017**
DGI (0–24)	16.3 (3.7)	18.2 (2.8)	2.0 (1.9)	**< 0.000**
6MWT (m)	282.2 (94.2)	316.7 (94.0)	34.5 (35.3)	**0.001**
10m Comfortable Speed (m/s)	0.71 (0.20)	0.78 (0.19)	0.1 (0.1)	**0.001**
10m Fastest Speed (m/s)	0.87 (0.27)	0.96 (0.25)	0.1 (0.1)	**0.003**
Peak Swing Angle (deg)	0.43 (6.9)	0.75 (6.9)	0.3 (2.6)	0.636
Maximum Toe Clearance (mm)	63.0 (29.2)	71.4 (24.8)	8.3 (18.1)	0.085
Minimum Toe Clearance (mm)	21.9 (17.0)	27.6 (24.3)	5.6 (12.2)	0.085
Maximum DF Angular Velocity (deg/s)	35.3 (31.6)	47.1 (38.8)	11.8 (16.8)	**0.013**
Hip Flexion (deg)	35.4 (8.8)	36.9 (7.3)	1.4 (6.3)	0.376
Hip Hike (cm)	8.2 (3.3)	7.5 (2.7)	−0.6 (2.3)	0.288
Hip Abduction (deg)	−7.9 (4.8)	−7.6 (6.1)	0.4 (3.6)	0.125
Knee Flexion (deg)	33.4 (17.6)	36.9 (17.2)	3.6 (5.3)	**0.015**
Paretic Step length (cm)	40.3 (14.3)	43.3 (12.2)	3.0 (4.7)	**0.021**
Paretic Swing (%)	40.6 (2.9)	39.3 (2.2)	0.8 (2.4)	**0.048**
Step Length Symmetry (%)	0.19 (0.13)	0.13 (0.10)	0.06 (0.1)	0.085
Stance Duration Symmetry (%)	0.17 (0.07)	0.15 (0.06)	0.02 (0.0)	0.123
Double Support Stance (%)	23.5 (4.0)	23.5 (3.2)	0.03 (2.2)	0.963
Heel-First Strikes (%steps)	31.3 (43.2)	44.8 (44.7)	13.5 (14.6)	**0.002**
HAI (%)* [six-visit]	7.0 (1.9)	9.6 (2.4)	2.7 (2.5)	0.05

Values in bold represent p < 0.05. HAI: human autonomy index. SIS-ADL: SIS score on ADL/IADL item.

**Table 4:** Comparison of change in functional outcomes against MCIDs.

Outcome	Δ(SD)	MCID/MDC
SIS-ADL (0–100)	6.9	9.2
SIS-Mobility (0–100)	**6.8**	5.9^[31]^
DGI (0–24)	**2.0**	1.9^[32]^
6MWT (m)	**34.4**	34.4^[33–34]^
10m Comfortable Speed (m/s)	0.07	0.18
10m Fastest Speed (m/s)	0.09	0.13
Knee Flexion (deg)	3.6	3.8

* Values in bold represent change greater than MCID/MDC.

## Data Availability

Information on this clinical trial (Clinical Trial Identifier: NCT04594837) can be found at: https://clinicaltrials.gov/study/NCT04594837.

## Abbreviations

3-D: three dimensional
AFO: ankle foot orthosis
FES: functional electrical stimulation
ROM: range of motion
DOF: degree of freedom
IADL/ADL: instrumented/activities of daily life
MMT: manual muscle test
6MWT: six-minute walk test
NYHA: New York Heart Association Functional Classification
